# Aromatic Residue Variations in the Central β‑Sheet Influence Stability and Activity of E. coli Glutaredoxin 3

**DOI:** 10.1021/acsomega.5c01938

**Published:** 2025-06-09

**Authors:** Mohammed Shazaly A. Elhassan, Trang Van Tran, ChangWoo Lee

**Affiliations:** † Department of Biomedical Science and Center for Bio-Nanomaterials, 37975 Daegu University, Gyeongsan 38453, South Korea; ‡ Phacogen Institute of Technology, Hanoi 10700, Vietnam

## Abstract

Bacterial glutaredoxin Grx3 is a Class I oxidoreductase with a canonical thioredoxin (Trx) fold but exhibits greater conformational flexibility than Trx due to the absence of one α-helix and one β-strand. In Escherichia coli Grx3 (EcGrx3), the adjacent β1 and β3 strands contain Tyr6 and Phe56, which interact with Arg46 in the α2 helix via hydrogen bonding and cation-π interactions, forming a stabilizing Tyr6-Arg46-Phe56 network. To investigate how aromatic residue variation at these sites affects EcGrx3 stability and activity, we introduced substitutions at Phe56 (F56A, F56S, F56I, F56Y, and F56W), Tyr6 (Y6F), and a double mutant (Y6F/F56Y). All mutants showed reduced melting temperatures and increased sensitivity to guanidinium chloride (GdmCl)-induced unfolding. Although F56Y and F56W can form cation−π interactions with Arg46, they exhibited the lowest thermal stability but distinct functional outcomes. F56Y retained wild-type-like activity and flexibility and was most resistant to chemical denaturation, while F56W, with higher α-helix content and rigidity, showed the highest catalytic efficiency but was highly GdmCl-sensitive. Aliphatic substitutions (F56A and F56I) caused moderate destabilization, while polar (F56S) and charged (F56E) mutants were more disruptive. Y6F significantly reduced both α-helix content and catalytic efficiency. These results demonstrate that both cation−π interactions and hydrophobic packing at position 56 are critical for EcGrx3 stability, with Phe56 providing optimal balance. Tyr6 stabilizes the β1−α2 interface via hydrogen bonding, and both residues are critical for α-helix formation. Together, these findings highlight how aromatic variation within the central β-sheet contributes to structural and functional adaptation in the Trx-fold superfamily.

## Graphical abstract

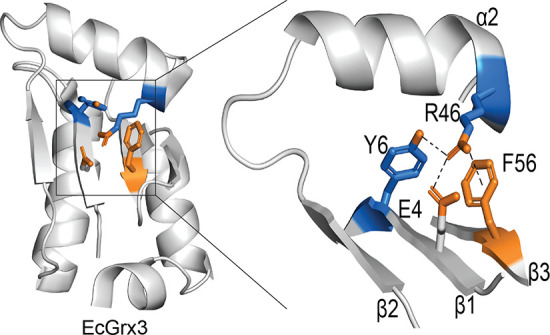

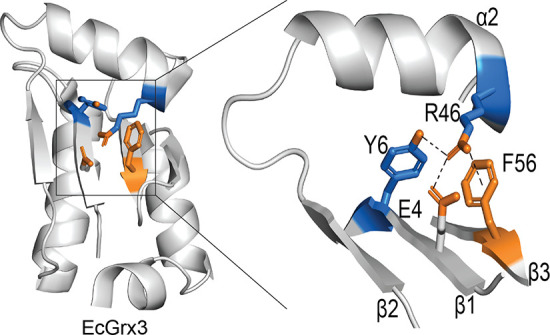

## 1 Introduction

Glutaredoxins (Grxs) are glutathione (GSH)-interacting members of the Trx-fold superfamily found across all domains of life.^1,2^ They play essential roles in maintaining cellular redox homeostasis, regulating oxidative stress, and facilitating protein folding and repair.^3,4^ Bacterial Grxs are categorized into at least four classes, with Class I and Class II being the most prevalent.^5^ Class I Grxs possess a monothiol or dithiol [CXXC/S] motif that support GSH-dependent oxidoreductase activity.^6,7^ A flexible loop near the N-terminal Cys facilitates its positioning for efficient electron transfer, playing a critical role in cellular antioxidant defense.^8,9^ In contrast, Class II Grxs contain the [CGFS] motif and function as Fe–S transferases rather than redox enzymes.^6,9^ A unique five-residue loop, anchored by a conserved N-terminal Lys (or Arg), orients GSH to support Fe–S cluster transfer.^9^

The Trx fold is an ancient protein architecture, characterized by a central β-sheet flanked by α-helices, that has persisted for over four billion years.^10^ The central β-sheet of EcTrx, which separates the hydrophobic core into aliphatic and aromatic clusters, has remained rigid throughout evolution,^11,12^ with hydrophobic interactions between the β2 and β4 strands essential in early folding events.^13^ Fragment analysis further suggests that β3, β2, and β4 in EcTrx form a cooperative folding subunit, preserving residual structure even in the unfolded state.^14^

Bacterial Grx3, a Class I oxidoreductase, adopts a canonical Trx fold with a four-stranded β-sheet surrounded by three α-helices but lacks one α-helix and one β-strand compared to Trx (Figure 1A,B).^15–18^ This structural modification reduces aromatic cluster hydrophobicity, with Tyr6 in β1 and Arg46 in α2 in EcGrx3 replacing Phe and Tyr/Phe at the corresponding Trx positions and stabilizing the β1-α2 interaction via hydrogen bonding.^15,19^ In Sphingomonas sp. Grx3 (SpGrx3), mutations at the corresponding residues (Y7F and R47F) led to protein aggregation at 40 °C and increased flexibility with reduced catalytic efficiency, respectively.^20^ Sequence comparisons show that Tyr6 (β1) and Phe56 (β3) are highly conserved across Grx3 orthologs—corresponding to Phe27 (β2) and Phe81 (β4) in EcTrx (Figure 1C). Phe56 is occasionally replaced by Tyr in mesophilic orthologs and by Trp in cold-adapted orthologs (psychrotrophic and psychrophilic), whereas Tyr6 is consistently retained. Trxs and Grxs contain few aromatic residues overall; Grxs have less than 1% Trp, while Phe and Tyr each exceed 2.5%.^18^ Notably, EcGrx3 lacks Trp entirely.

**Figure 1:**
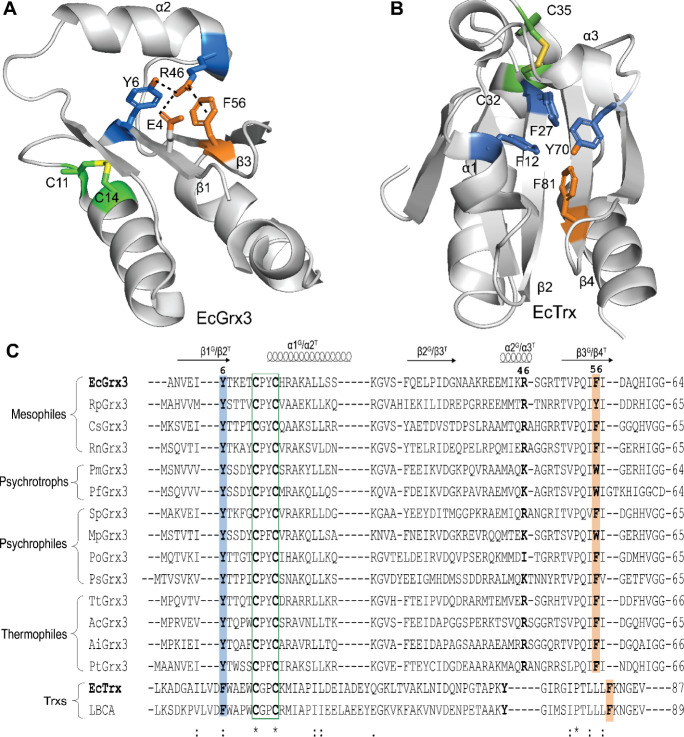
Structure and sequence comparison of bacterial Grx3 and Trx orthologs. (A) Structure of EcGrx3 showing interactions among β1 (Glu4, Tyr6), α2 (Arg46), and β3 (Phe56). (B) Structure of EcTrx showing the tetrahedral arrangement of aromatic residues (Phe12, Phe27, Tyr70, Phe81). Phe56 and its corresponding Phe81 are shown in orange, and active-site Cys residues are shown in green. (C) Multiple sequence alignment of bacterial Grx3 and Trx orthologs. The active site motif is boxed in green. α-Helices and β-strands are labeled with superscripts G (Grx3) and T (Trx). Conserved aromatic residues in the β1/β3 of Grx3 and β2/β4 of Trx are highlighted in blue and orange, respectively. **Mesophilic Grx3**: EcGrx3 (E. coli, PDB ID: 1FOV), RpGrx3 (Ralstonia pseudosolanacearum, NCBI ID: AGH82898.1), CsGrx3 (Cereibacter sphaeroides, NCBI ID: QHA14359.1), RnGrx3 (Rheinheimera nanhaiensis, NCBI ID: WP_008217797.1); **Psychrotrophic Grx3**: PmGrx3 (Pseudomonas mandelii, NCBI ID: TWS12334.1), PfGrx3 (Pseudomonas fluorescens, NCBI ID: WP_038445308.1); **Psychrophilic Grx3**: SpGrx3 (Sphingomonas sp., NCBI ID: WP_157026847.1), MpGrx3 (Marinomonas primoryensis, NCBI ID: WP_392386135.1), PoGrx3 (Polaromonas sp., NCBI ID: UUZ77350.1), PsGrx3 (Psychrobacter sp., NCBI ID: GI_MW245063); **Thermophilic Grx3**: TtGrx3 (Thermochromatium tepidum, NCBI ID: WP_153975213.1), AcGrx3 (Acidisphaera sp. *L21*, NCBI ID: WP_158742549.1), AiGrx3 (Acidibrevibacterium fodinaquatile, NCBI ID: WP_114911700.1), PtGrx3 (Phomidium tenue, NCBI ID: WP_191130001.1). **Trx orthologs**: EcTrx (E. coli, PDB ID: 2TRX), LBCA (Last Bacterial Common Ancestor, PDB ID: 4BA7).

To examine the functional significance of aromatic residues in the central β-sheet, this study investigates how variation at Phe56 and the conservation of Tyr6 in EcGrx3 affect structural stability and oxidoreductase activity. Structural analysis shows that Arg46 forms a salt bridge with Glu4, a hydrogen bond with Tyr6, and a cation−π interaction with Phe56, forming a stabilizing interaction network at the β1-α2-β3 interface (Figure 1A). To probe the role of Phe56, we introduced aliphatic (F56A, F56I), polar (F56S), charged (F56E), and aromatic (F56Y, F56W) substitutions; F56Y and F56W reflect naturally occurring variants. We also generated the Y6F mutant to reinstate Phe at β1, as in EcTrx, and the Y6F/F56Y double mutant to reverse the WT Tyr6-Phe56 configuration. This study further explores how aromatic residue variation contributes to temperature adaptation across Grx3 orthologs.

## 2 Materials and Methods

### 2.1 Gene Cloning and Site-Directed Mutagenesis

The *grxC* (*ecgrx3*) gene was synthesized by Bioneers (Daejeon, South Korea) based on the E. coli K-12 *grxC* sequence (NCBI ID: 948132). Site-directed mutagenesis was performed using the pET28-*ecgrx3* construct as a template, with primers listed in Table S1. PCR reactions (20 μL) contained 9 μL PCR-grade water, 5 μL dNTPs (2.0 mM), 2 μL 10× Pfu buffer, 2 μL template, 1 μL primer, and 1 μL Pfu-forte polymerase. Thermal cycling conditions were an initial denaturation at 95 °C for 2 min, 25 cycles of 95 °C for 30 s, 55–60 °C for 30 s, and 72 °C for 6 min; followed by a final extension at 72 °C for 10 min. PCR products were digested with DpnI (Enzynomics, Daejeon, South Korea) at 37 °C for 1 h to digest parental plasmids. The digested products were transformed into E. coli BL21­(DE3) for protein expression. All constructions were verified by Sanger sequencing.

### 2.2 Bioinformatics

Structural models were generated using the AlphaFold2 within ChimeraX (version 1.9).^21^ Model confidence was evaluated using the pLDDT (predicted Local Distance Difference Test) metric, with scores above 95 for EcGrx3 mutants, indicating high structural reliability.^22^ Amino acid interactions and structural features were analyzed and visualized using PyMOL (The PyMOL Molecular Graphics System, Version 2.0, Schrödinger, LLC.) and ChimeraX.^23^ Protein sequences were aligned using Clustal Omega.^24^ Cation-π and π-π interactions were identified using the Capture and the Residue Interaction Networks web servers.^25,26^

### 2.3 Protein Expression and Purification

E. coli BL21­(DE3) cells were grown overnight in Luria–Bertani (LB) medium containing 100 μg/mL kanamycin, starting from a single colony. The overnight culture was inoculated into 200 mL of fresh LB medium and incubated at 37 °C until the optical density at 600 nm reached 0.6–0.8. Protein expression was induced by adding isopropyl β-d-1-thio-galactopyranoside (IPTG) to a final concentration of 1 mM, followed by incubation at 30 °C for 24 h. Cells were harvested by centrifugation at 4000 rpm for 15 min at 4 °C and washed with buffer A (50 mM Tris·HCl, 50 mM NaCl, 5 mM imidazole, 0.1 mM EDTA, pH 8.0). The cell pellets were resuspended in buffer A and sonicated on ice. The lysate was centrifuged at 10,000 × g for 30 min at 4 °C, and the supernatant was collected. The supernatant was loaded onto a 1 mL HisTrap column (Cytiva, Marlborough, MA, USA) pre-equilibrated with buffer A. Nonspecific proteins were removed by washing with buffer A supplemented with 20 mM imidazole. EcGrx3 proteins were eluted using buffer B (buffer A with 300 mM imidazole). Fractions containing EcGrx3 were further purified using a 1 mL Capto Q column on an AKTA go system (Cytiva), pre-equilibrated with buffer C (50 mM Tris·HCl, 50 mM NaCl, pH 8.0). Fractions containing the target proteins were desalted using a HiTrap desalting column and transferred into buffer D (100 mM Tris·HCl, pH 8.0) with 5% glycerol. Purified proteins were kept at −80 °C. The molecular weights (MWs) of the proteins were estimated by size-exclusion chromatography using a Superdex 200 prep grade XK16 column in buffer C. Calibration was performed using a protein standard mix containing β-amylase (200 kDa), alcohol dehydrogenase (150 kDa), albumin (66 kDa), carbonic anhydrase (29 kDa), and cytochrome c (12.4 kDa).

### 2.4 Protein Thermal Shift Analysis

A thermal shift assay was performed using SYPRO Orange dye on an Applied Biosystems StepOnePlus real-time PCR instrument (Thermo Fisher Scientific, Waltham, MA, USA) in continuous ramp mode at a rate of 1% per min, from 25 to 99 °C.^27^ Protein samples were prepared at a final concentration of 0.3 mg/mL in buffer D with 3× SYPRO Orange dye, in a total reaction volume of 20 μL. The melting temperature (*T*_m_) was determined using Protein Thermal Shift software (version 1.4; Thermo Fisher Scientific).

### 2.5 Conformational Stability Assessment

Equilibrium unfolding curves of EcGrx3 wild-type (WT) and mutants were measured using fluorescence spectroscopy in the presence of GdmCl on a Scinco FS-2 fluorescence spectrometer (Seoul, South Korea). As EcGrx3 lacks Trp residues, Tyr fluorescence was monitored for the WT and all mutants except F56W (excitation at 275 nm). For the F56W mutant, Trp fluorescence was used (excitation at 285 nm). Protein samples (80 μg) were incubated with increasing concentrations of GdmCl (0–6 M) in buffer E (100 mM potassium phosphate, 150 mM NaCl, pH 7.0) at 25 °C for 20 min. The unfolding process was analyzed by calculating the equilibrium constant (*K*_eq_) for each GdmCl concentration, and the unfolding free energy ( ${\Delta G}_{H_{2}O}^{0\prime }$ ) as determined using the equation:^28^

$${\Delta G}_{H_{2}O}^{0\prime }=-RT\ln\left(K_{\mathrm{eq}}\right)$$

where R is the gas constant (8.314 J·K^–1^·mol^–1^) and T is the temperature in Kelvin (298 K).^29^

### 2.6 Fluorescence Spectroscopy Analysis

Acrylamide quenching was assessed by incubating 100 μg of each protein in buffer D at 25 °C with increasing acrylamide concentrations (0–0.25 M). After a 5 min incubation, fluorescence measurements were recorded using a Scinco FS-2 fluorescence spectrometer. Fluorescence quenching was analyzed by plotting the ratio of fluorescence intensities (F_0_/F) against the acrylamide concentration [Q] to generate Stern–Volmer plots. The Stern–Volmer constant (*K*_SV_) was calculated by fitting the linear region of the plot to the equation:^30^

$$F_{0}/F=1+K_{\mathrm{SV}}\left[Q\right]$$

where F_0_ and F are the fluorescence intensities in the absence and presence of acrylamide, respectively, and [Q] is the acrylamide concentration.

### 2.7 Far-UV Circular Dichroism (CD) Analysis

CD spectra were measured in the far-UV range (190–260 nm) at 25 °C using a JASCO J-1500 spectropolarimeter (Tokyo, Japan) at the Korea Basic Science Institute (Ochang, South Korea). Protein samples (1 mg/mL, 200 μL) were prepared in buffer C and incubated at 4 °C for 1 h prior to measurement. Spectra were recorded using a 1 mm path length cuvette with a bandwidth of 1.0 nm, a data pitch of 0.1 nm, and a scanning speed of 100 nm/min. The scanning mode was continuous, and each spectrum was obtained by averaging three scans with a 1-s integration time. CD spectra were presented as mean residual ellipticity (mdeg) versus wavelength. Secondary structure content was analyzed using the BeStSel server for experimental CD data^31^ and STRIDE for AlphaFold2-predicted models.^32^

### 2.8 Activity and Kinetics Analysis

Grx3 activity was measured using a hydroxyethyl disulfide (HED) reduction assay, as previously described.^33,34^ Reactions were conducted in buffer D containing 1 mM GSH, 0.4 mM NADPH, 2 mM EDTA, 0.1 mg/mL bovine serum albumin, and 0.4 μg yeast glutathione reductase (Sigma). An 800-μL aliquot of this mixture was combined with 2.5 mM HED (Sigma) and 13.8 μg of EcGrx3 protein. NADPH oxidation was monitored at 340 nm for 5 min using a Shimadzu UV-1800 spectrophotometer (Kyoto, Japan). Enzyme kinetics were analyzed by measuring enzyme activity across a range of GSH concentrations (0.05–1 mM) at the optimal temperature for WT and each mutant. Kinetic parameters (*K*_m_, *V*_max_, and *k*_cat_) were derived from Lineweaver–Burk plots.

### 2.9 Molecular Dynamics (MD) Simulations

MD simulations of EcGrx3 WT and its aromatic mutants (F56W, F56Y, Y6F/F56Y) were performed using Maestro 12.0 (Schrödinger, LLC, New York, NY, USA). The OPLS4 force field^35^ and SPC explicit solvent model^36^ were used to define the energetic parameters. Protein structures were prepared by removing water molecules and optimizing geometries using the Protein Preparation Wizard. To maintain electrical neutrality, Na^+^ and Cl^–^ ions were added by replacing water molecules. The systems were energy-minimized to relieve steric clashes and equilibrated under positional restraints at 300 K. Production simulations were performed at BioCode Ltd. (Liverpool, Merseyside, UK) for 100 ns under an NPT ensemble using a 2 fs time step. The root-mean-square deviation (RMSD) of C_α_ atoms was calculated to assess global conformational stability, and the root-mean-square fluctuation (RMSF) of C_α_ atoms was analyzed to evaluate local, residue-level flexibility.

## 3 Results

### 3.1 Expression and Purification

The N-terminal 6 × His-tagged recombinant EcGrx3 WT and its mutants were successfully expressed as soluble proteins in E. coli BL21­(DE3). The proteins were purified to homogeneity using a combination of HisTrap nickel-affinity chromatography, Capto Q anion-exchange chromatography, and size-exclusion chromatography using Superdex 200. SDS-PAGE analysis showed a single band for both the WT and mutant proteins (Figure S1). Size-exclusion chromatography indicated that WT and mutants eluted as monomers, with an MW of 12.5 kDa (Figure S2).

### 3.2 Protein Thermal Shift Analysis

*T*_m_ values, indicating the temperature at which 50% of the protein is denatured, were determined for EcGrx3 WT and mutants using SYPRO Orange-based thermal shift analysis (Table 1 and Figure S3). All Phe56 mutants exhibited lower *T*_m_ values than WT (66.3 °C). Aliphatic substitutions caused moderate destabilization, with similar *T*_m_ values for F56A (60.2 °C) and F56I (59.0 °C), suggesting that hydrophobicity alone does not ensure thermal stability. In contrast, the charged F56E (56.5 °C) and polar F56S (45.0 °C) led to greater destabilization. The largest reductions were observed in F56W (43.7 °C) and F56Y (42.5 °C), likely due to steric effects and altered cation-π interactions with Arg46. Y6F lowered the *T*_m_ to 54.1 °C, while the Y6F/F56Y double mutant further reduced it to 42.3 °C—similar to F56Y alone—implying that the F56Y substitution primarily drives the loss in stability. These results underscore that Phe56 is essential for EcGrx3 stability through both cation-π interactions with Arg46 and hydrophobic packing, while Tyr6 supports the β1-α2 interaction through hydrogen bonding.

**Table 1:** *T*_m_ Values of EcGrx3 WT and Mutants^a^

	*T*_m_ (°C)
WT	66.3 ± 0.02
Y6F	54.1 ± 0.02
F56A	60.2 ± 0.01
F56E	56.5 ± 0.03
F56I	59.0 ± 0.01
F56S	45.0 ± 0.02
F56W	43.7 ± 0.01
F56Y	42.5 ± 0.03
Y6F/F56Y	42.3 ± 0.02

a Data represent the means ± S.D. from three independent experiments.

### 3.3 Conformational Stability Evaluation

The conformational stability of EcGrx3 WT and mutants was assessed by GdmCl-induced unfolding, monitored by fluorescence spectroscopy at 25 °C. Fluorescence profiles varied by mutant: Y6F showed minimal signal, confirming Tyr6 as the primary fluorophore in WT and Tyr-retaining mutants (Figure S4). All mutants showed denaturation curve shifts toward lower GdmCl concentrations relative to WT (3.3 M) (Table S2 and Figure 2), with decreasing [D]_1/2_ values as follows: F56Y (2.9 M) > F56A (2.6 M) > F56I (2.3 M) > F56S (2.1 M) > Y6F/F56Y (1.9M) > F56E (1.7 M) > F56W (1.3 M). Aliphatic substitutions (F56A, F56I) caused moderate destabilization, suggesting that hydrophobic packing at position 56—retained to some extent in these mutants—helps preserve chemical stability. In contrast, polar (F56S) and charged (F56E) substitutions were more disruptive. F56Y was the most resistant to chemical denaturation among the mutants, despite its low *T*_m_, indicating that chemical and thermal unfolding follow distinct mechanisms. Conversely, F56W, which also had a low *T*_m_, was the most GdmCl-sensitive, reflecting structural instability under both conditions. These results suggest that Phe56 stabilizes EcGrx3 through hydrophobic packing, which enhances chemical stability, and cation−π interactions, which support thermal resistance—highlighting the differential roles of side-chain properties in chemical versus thermal unfolding.

**Figure 2:**
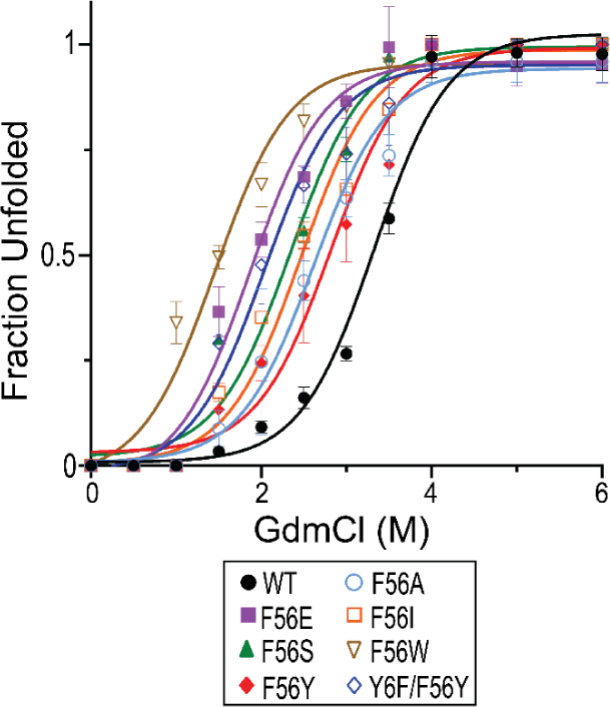
GdmCl-induced unfolding of EcGrx3 WT and mutants. Fluorescence spectra were measured after incubating the proteins with different concentrations of GdmCl (0–6 M) at 25 °C for 20 min. Data represent the mean ± SD of three independent experiments.

### 3.4 Conformational Flexibility Assessment

The conformational flexibility of EcGrx3 WT and mutants was evaluated using acrylamide-induced quenching of intrinsic fluorescence at 25 °C. The flexibility ranking was: F56S > F56A > F56I > F56E > Y6F/F56Y > F56Y > WT > F56W (Table S3 and Figure 3). Substitutions with small side chains, such as F56S and F56A, increased flexibility by reducing steric constraints, while the bulkier F56I showed slightly less flexibility. In contrast, F56E exhibited intermediate flexibility, likely due to the influence of charge on backbone dynamics. Aromatic mutants displayed overall rigidity, with flexibility decreasing in the order F56Y > WT > F56W. F56W was the most rigid, likely due to the large indole ring, which enhances cation−π interactions with Arg46, together with side-chain bulk, stabilize the local structure. In contrast, the unpaired hydroxyl group of Tyr56 may reduce packing efficiency, contributing to its slightly higher flexibility compared to WT. The Y6F/F56Y double mutant also showed intermediate flexibility—greater than F56Y—reflecting an additive effect of Tyr6 loss and Tyr56 substitution. These findings underscore that both cation−π interactions and side-chain size at position 56 cooperatively regulate local conformational rigidity.

**Figure 3:**
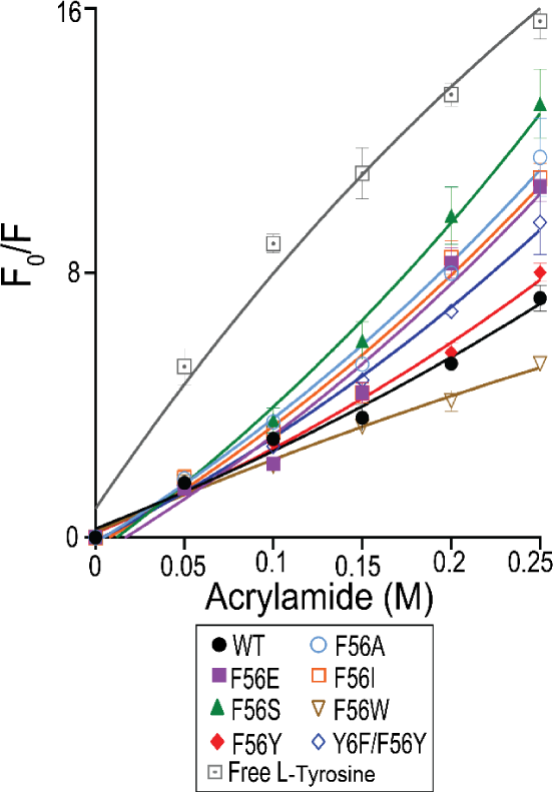
Acrylamide Stern–Volmer plot of EcGrx3 WT and mutants. The plot shows the maximum fluorescence intensity (F_0_) in the absence of acrylamide and at increasing acrylamide concentrations (0–0.25 M). Data represent the mean ± SD from three independent experiments.

### 3.5 Secondary Structure Analysis

The secondary structure of EcGrx3 WT and its mutants was assessed by far-UV CD spectroscopy at 25 °C (Table S4 and Figure 4). The α-helix content followed the order: F56W (57%) > WT (50%) > F56Y (42%) > F56A (35%) > F56I (34%) > F56S ≈ Y6F/F56Y (18%) > F56E (10%) > Y6F (8%). All Phe56 mutants except F56W showed reduced helicity relative to WT. Among aromatic mutants, the trend generally reflects the strength of cation-π interactions—with Trp strongest—though hydrophobicity also plays a role, as seen in the higher helicity of Phe compared to Tyr. Y6F showed the lowest helicity due to the loss of β1-α2 hydrogen bonding, while the Y6F/F56Y double mutant partially restored it. Among nonaromatic mutants, F56A and F56I retained intermediate helicity, indicating partial preservation of secondary structure, while F56S and F56E caused substantial loss. These results suggest that both aromaticity and hydrophobicity at position 56 are important for α-helix stability, with Phe56 uniquely maintaining high helicity without compromising overall stability or flexibility—supporting its conservation across Grx3 orthologs. By contrast, STRIDE analysis of AlphaFold2-predicted models estimated a uniform ∼47% helicity across all mutants, underscoring the limitations of static predictions in capturing experimental variation.

**Figure 4:**
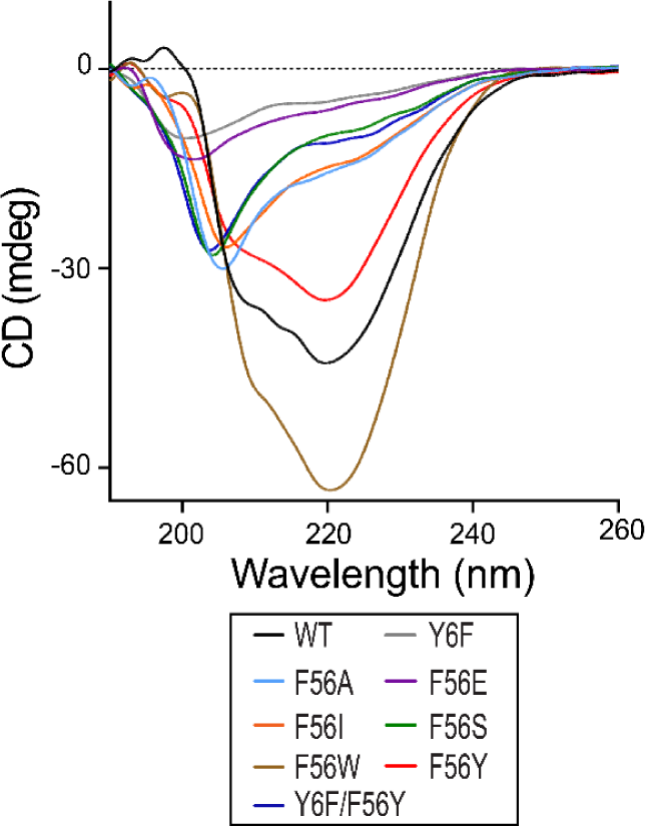
Far UV-CD spectra of EcGrx3 WT and mutants. The CD spectra was measured at 25 °C after a 1-h incubation at 4 °C.

### 3.6 Enzyme Kinetics

Kinetic parameters of EcGrx3 WT and its mutants were determined using the HED reduction assay with GSH and NADPH at their respective optimal temperatures (Table 2, Figures S5 and S6). Aromatic mutants (F56W, F56Y, Y6F/F56Y) showed improved substrate affinity, with lower *K*_m_ values than WT (2.43 mM). F56W exhibited the highest catalytic efficiency (2.05 s^–1^mM^–1^), due to both a reduced *K*_m_ (1.49 mM) and increased *k*_cat_ (3.0 s^–1^), suggesting a stabilizing role for the bulky indole ring. F56Y and Y6F/F56Y had near-WT efficiency. In contrast, nonaromatic mutants (F56A, F56S) showed reduced substrate affinity and lower efficiencies (*K*_m_ = 3.38 and 3.13 mM; *k*_cat_/*K*_m_ = 0.55 and 0.43 s^–1^mM^–1^). F56E retained a WT-like *K*_m_ but had a lower *k*_cat_, while F56I preserved both parameters, likely through hydrophobic compensation. Y6F also showed reduced efficiency despite a lower *K*_m_, suggesting a role for Tyr6 in turnover via β1-α2 hydrogen bonding. These findings indicate that aromatic residues at position 56—especially Trp—enhance activity by promoting a favorable active-site conformation through local structural rigidity, while loss of aromaticity or hydrogen bonding impairs catalysis by weakening active-site support.

**Table 2:** Kinetic Parameters of EcGrx3 WT and Mutants^a^

	***K***_m_ (mM)	***k***_cat_ (s^–1^)	***k***_cat_/***K***_m_ (s^–1^·mM^–1^)
WT	2.43 ± 0.04	1.8 ± 0.01	0.79 ± 0.01
Y6F	1.95 ± 0.03	1.1 ± 0.02	0.57 ± 0.02
F56A	3.38 ± 0.04	1.8 ± 0.03	0.55 ± 0.07
F56E	2.58 ± 0.02	1.3 ± 0.01	0.50 ± 0.03
F56I	2.35 ± 0.13	1.7 ± 0.13	0.79 ± 0.01
F56S	3.13 ± 0.14	1.3 ± 0.07	0.43 ± 0.01
F56W	1.49 ± 0.01	3.0 ± 0.01	2.05 ± 0.01
F56Y	2.08 ± 0.01	1.6 ± 0.04	0.77 ± 0.01
Y6F/F56Y	2.30 ± 0.04	2.0 ± 0.03	0.87 ± 0.20

a The data are expressed as means ± S.D. from three experiments.

### 3.7 MD Simulations

To better understand the structural basis for the divergent stability and activity of aromatic mutants, MD simulations were performed on the WT protein and the F56W, F56Y, and Y6F/F56Y mutants, which form cation−π interactions with Arg46 (Figure 5). RMSD analysis showed that WT and F56Y maintained relatively stable conformations throughout the 100 ns simulation (∼0.7–1.2 Å), while F56W and Y6F/F56Y displayed larger fluctuations (∼1.0–2.0 Å). RMSF analysis revealed distinct flexibility patterns among the mutants. While all showed increased flexibility at the *N*- and C-termini relative to WT, F56W and Y6F/F56Y exhibited the largest fluctuations. F56W also elevated mobility in the β2-α2 loop, α2 helix, and β3 loop, likely due to steric effects from the bulky Trp side chain. In contrast, F56Y remained relatively rigid overall, especially in the β1-α1 and β2-α2 loops, despite some increased flexibility in α2. The Y6F/F56Y double mutant showed the most extensive destabilization, with increased flexibility in β1, β3, β2-α2 loop, and α2 helix, reflecting disruption of the Tyr6 hydrogen bond and altered packing around Tyr56. Together, these MD simulations support the experimental findings by showing that F56W exhibits localized flexibility despite overall rigidity, F56Y maintains relative rigidity and chemical stability, and Y6F/F56Y is the most destabilized, consistent with its reduced thermal and conformational stability.

**Figure 5:**
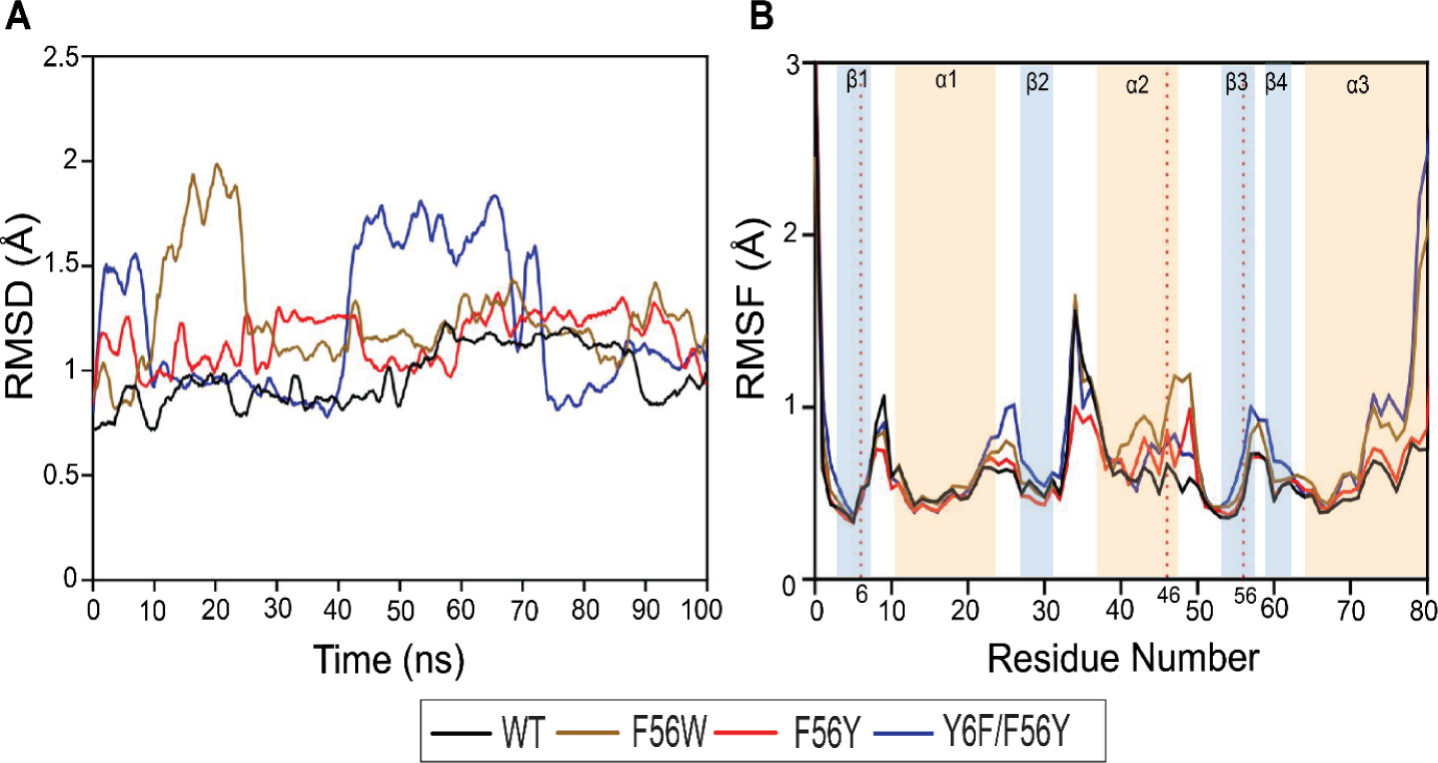
RMSD and RMSF profiles of EcGrx3 WT and mutants (F56W, F56Y, and Y6F/F56Y) from a 100 ns MD simulation. (A) C_α_ RMSD over time. (B) C_α_ RMSF per residue, with secondary structure elements annotated.

## 4 Discussion

The Trx fold is a highly conserved structural motif found in enzymes that catalyze disulfide bond formation and isomerization.^37–39^ In EcTrx, the central β-sheet supports a tetrahedral aromatic cluster formed by Phe12 (α1), Phe27 (β2), Tyr70 (α3), and Phe81 (β4) (Figure 1B).^40^ This β-sheet is evolutionarily conserved and structurally rigid across Trx orthologs, while the surrounding helices exhibit variable flexibility: α3 tend to be more flexible, whereas α4 becomes more rigid.^12,41^ Hydrophobic interactions between β2 and β4 link the βαβαβ and ββα folding units,^13,42^ with Phe27 in β2 playing an important role in Trx stability—even in psychrophilic variants.^43,44^

Grx3 retains the canonical Trx fold but has diverged functionally to support GSH-dependent oxidoreductase activity, in contrast to Trx’s disulfide-based redox cycling.^1^ A defining feature of Grx3 is its β1-α2-β3 region, which include a Tyr6-Arg46-Phe56 triad. Arg46 forms a hydrogen bond with Tyr6 and a cation-π interaction with Phe56, both of which contribute to local stabilization.^19,45^ Grx3 also features a conserved β3-β4 hydrogen bond and more flexible loops, along with an inverted electrostatic potential near the active site that modulates substrate specificity.^46^

Our results underscore the critical role of Phe56 in maintaining EcGrx3 structural integrity through both a cation-π interaction with Arg46 and hydrophobic packing. Although F56Y and F56W also form cation-π interactions (Figure S7), their divergent side-chain properties alter the balance between stability and activity. F56Y retained WT-like catalytic efficiency and flexibility, despite its low thermal stability, and resisted GdmCl-induced unfolding, indicating different mechanisms for thermal and chemical denaturation. In contrast, F56W exhibited the highest catalytic efficiency, α-helix content, and rigidity, but was unstable under both denaturing conditions—reflecting a trade-off between enhanced activity and structural stability. MD simulations supported these findings: F56Y maintained structural rigidity consistent with its chemical stability; F56W, though rigid overall, showed localized flexibility likely due to steric effects; and Y6F/F56Y exhibited the greatest conformational fluctuations. These findings also suggest that side-chain hydrophobicity can partially compensate for the loss of aromaticity, as seen in F56I, which maintained catalytic efficiency and intermediate helicity. In contrast, smaller or polar substitutions (F56A, F56S, F56E) disrupted hydrophobic packing and compromised both stability and activity, underscoring the dual importance of aromaticity and hydrophobicity at position 56.

While the intrinsic strength of cation−π interactions generally follows the order Trp > Tyr > Phe,^47,48^ our data suggest that Phe56 provides optimal stabilization in EcGrx3. Its nonpolar nature may allow tighter packing and more favorable positioning relative to Arg46 than Tyr, whose hydroxyl group can disrupt local hydrophobicity. Thus, although Trp and Tyr can form stronger cation−π interactions in principle, Phe provides a more favorable balance between structural stability and function in this context.

Tyr6 also contributes to stability by forming a hydrogen bond with Arg46, stabilizing the β1-α2 interface and supporting α-helix formation. The detrimental effects of the Y6F/F56Y double mutant—resulting from the loss of hydrogen bonding and reduced thermal stability—support the notion that this configuration is not favored in natural Grx3 orthologs.

These findings also provide insights into cold adaptation. The F56W mutant exhibited a reduced optimal temperature of 30 °C (Figure S5), compared to 50 °C for WT, similar to the psychrophilic PsGrx3, which functions optimally at 25 °C despite retaining Phe at position 56.^49^ Although Trp confers a low *T*_m_ and enhances catalytic activity, it is rare (<7%) among ∼5000 Grx3 sequences analyzed (data not shown). In cold-adapted orthologs with Trp (e.g., PmGrx3 and MpGrx3), it co-occurs with substitutions such as Glu4-to-Val and Arg46-to-Lys, which may increase flexibility while preserving cation-π interactions (Figure S8). These observations suggest that Trp or Tyr at position 56 can facilitate cold adaptation by enhancing flexibility and catalytic activity—though often at the expense of structural stability. This trend contrasts with α/β-hydrolases, where Trp is typically found in mesophilic and psychrotrophic orthologs to support packing, while Tyr is more common in hyperthermophilic and psychrophilic counterparts—likely due to reduced hydrophobicity of aromatic residues at temperature extremes and Tyr’s capacity for hydrogen bonding.^50,51^

Class I Grx proteins in humans, plants, and parasites typically retain a Phe-Phe pair in β1 and β3—along with a hydrophobic α3 helix (EcGrx3 α2)—to stabilize the β-sheet stability via π-π stacking (Figure 6). In contrast, Class II Grxs more commonly feature a Phe-Tyr pair, which is absent in bacterial Class I Grx3 orthologs. Additional stabilizing interactions, such as hydrogen bonding (e.g., Ser67-Tyr76 in SpGrx4),^52^ or π-π stacking (e.g., Tyr103-Tyr113 in hGrx5), help accommodate the Tyr residue (Figure 6).

**Figure 6:**
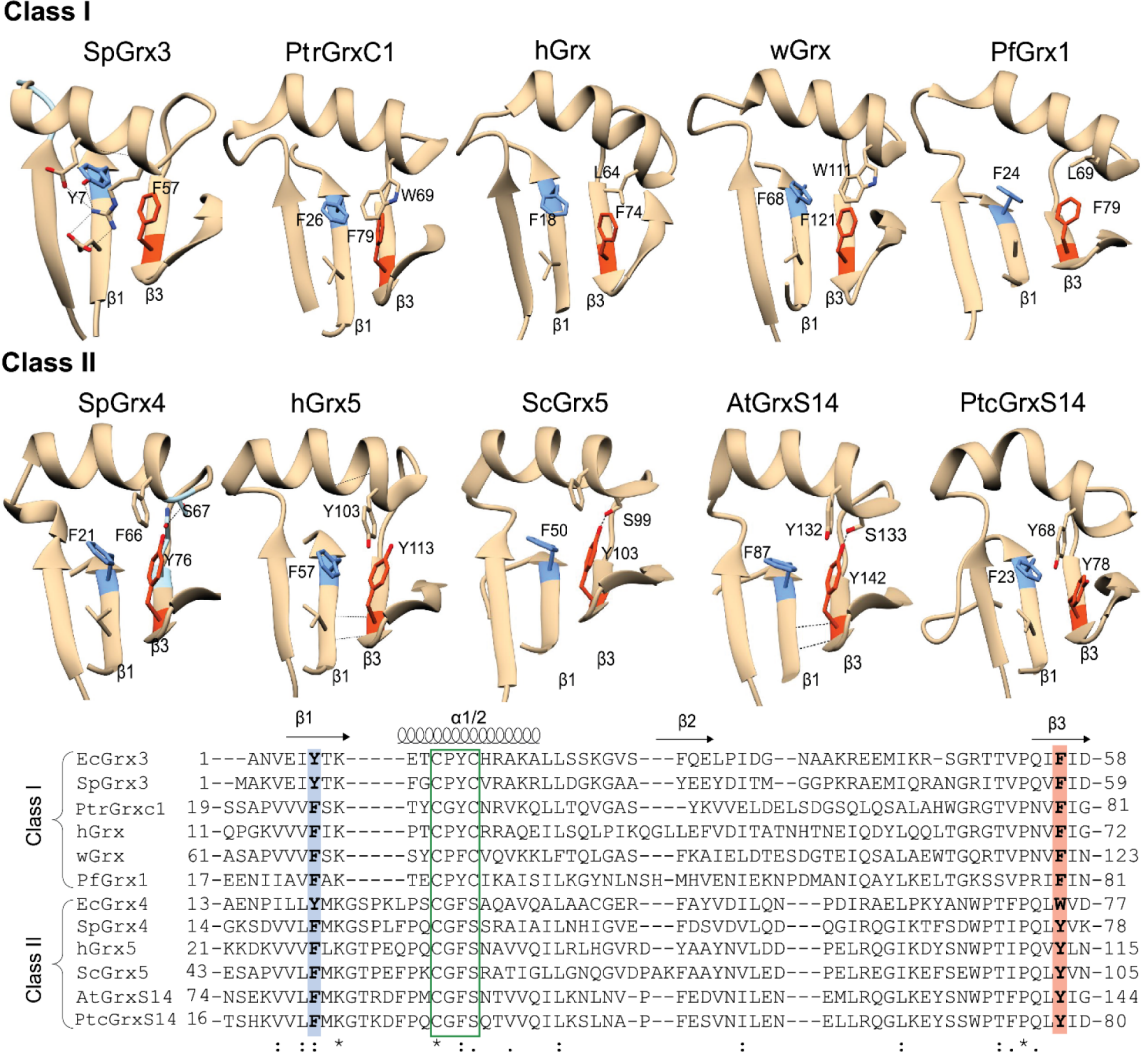
Comparison of the aromatic residue distribution in the central β-sheet of class I and class II Grx members and sequence comparison, highlighting β1and β3. β-strands are labeled as β1, β2 and β3, for all proteins to ensure uniform comparison. The structures shown include EcGrx3 (PDB ID: 1FOV), SpGrx3 (NCBI ID: WP_010217562.1), PtrGrxC1 (PDB ID: 1Z7P), hGrx (PDB ID: 1JHB), wGrx (PDB ID: 5ZVL), PfGrx1 (PDB ID: 4MZC), EcGrx4 (NCBI ID: WP_001764546.1), SpGrx4 (NCBI ID: WP_010164075.1), hGrx5 (PDB ID: 2MMZ), ScGrx5 (PDB ID: 3GX8), AtGrxS14 (PDB ID: 3IPZ), and PtcGrxS14 (PDB ID: 2LKU). Abbreviations: *Sp*, Sphingomonas sp.; *Ptr*, Populus tremula; h, human*;* w, wheat (Triticum aestivum); *Pf*, Plasmodium falciparum; *Sc*, Saccharomyces cerevisiae; *At*, Arabidopsis thaliana *; Ptc*, Populus trichocarpa.

## 5 Conclusions

Our findings demonstrate that the essential roles of Phe56 and Tyr6 in EcGrx3 stability and activity. The Tyr6-Arg46-Phe56 network stabilizes the β1-α2-β3 interface through hydrogen bonding and cation-π interactions, supporting α-helix formation and structural integrity. Substitutions at Phe56 revealed that side-chain properties fine-tune the balance between stability and activity: Trp enhanced catalytic efficiency but reduced both thermal and chemical stability, while Tyr maintained flexibility with moderate activity and improved resistance to chemical denaturation. These findings show how aromatic residue variation modulates the structural and functional adaptability of Grx3 within the Trx-fold superfamily.

## Supplementary Material



## Notes

Information for EcGrx3 WT and mutants, including PCR primers for site-directed mutagenesis, stability parameters, inverse Stern–Volmer quenching constants, secondary structure composition, SDS-PAGE for protein purity, size-exclusion chromatography for MW estimation, thermal denaturation curves, intrinsic fluorescence measurements, apparent optimum temperatures, Lineweaver–Burk plots, cation−π interactions of F56W and F56Y mutants, and structural comparisons of mesophilic, psychrotrophic, and psychrophilic Grx3 orthologs (PDF)

## Abbreviations

EDTA: Ethylenediaminetetraacetic acid
NADPH: Nicotinamide adenine dinucleotide phosphate
dNTPs: Deoxynucleotide triphosphates
HED: hydroxyethyl disulfide

## References

[1] Ogata F. T., Branco V., Vale F. F., Coppo L. (2021). Glutaredoxin: Discovery,
redox defense and much more. Redox Biol..

[2] Sevilla F., Marti M. C., De brasi-Velasco S., Jimenez A. (2023). Redox regulation, thioredoxins,
and glutaredoxins in retrograde signalling and gene transcription. J. Exp. Bot..

[3] Elgan T. H., Planson A. G., Beckwith J., Guntert P., Berndt K. D. (2010). Determinants
of activity in glutaredoxins: An *in vitro* evolved
Grx1-like variant of Escherichia coli Grx3. Biochem. J..

[4] Daniel T., Faruq H. M., Laura
Magdalena J., Manuela G., Christopher
Horst L. (2020). Role of GSH and iron-sulfur glutaredoxins in iron metabolism-Review. Molecules.

[5] Couturier J., Jacquot J.-P., Rouhier N. (2013). Toward a refined classification
of
class I dithiol glutaredoxins from poplar: Biochemical basis for the
definition of two subclasses. Front. Plant Sci..

[6] Liedgens L., Zimmermann J., Wäschenbach L., Geissel F., Laporte H., Gohlke H., Morgan B., Deponte M. (2020). Quantitative assessment
of the determinant structural differences between redox-active and
inactive glutaredoxins. Nat. Commun..

[7] Chai Y. C., Mieyal J. J. (2023). Glutathione and
glutaredoxin-key players in cellular
redox homeostasis and signaling. Antioxidants.

[8] Liedgens, L. ; Deponte, M. The catalytic mechanism of glutaredoxins. In Glutathione; CRC Press: 2018, pp. 251–261.

[9] Trnka D., Engelke A. D., Gellert M., Moseler A., Hossain M. F., Lindenberg T. T., Pedroletti L., Odermatt B., de Souza J. V., Bronowska A. K. (2020). Molecular basis for the distinct functions
of redox-active and FeS-transfering glutaredoxins. Nat. Commun..

[10] Romero-Romero M. L., Risso V. A., Martinez-Rodriguez S., Ibarra-Molero B., Sanchez-Ruiz J. M. (2016). Engineering ancestral protein hyperstability. Biochem. J..

[11] Campitelli P., Modi T., Kumar S., Ozkan S. B. (2020). The role of conformational
dynamics and allostery in modulating protein evolution. Annu. Rev. Biophys..

[12] Modi T., Huihui J., Ghosh K., Ozkan S. B. (2018). Ancient thioredoxins
evolved to modern-day stability–function requirement by altering
native state ensemble. Philos. Trans. R. Soc.
London, B Biol. Sci..

[13] Tasayco M. L., Fuchs J., Yang X. M., Dyalram D., Georgescu R. E. (2000). Interaction
between two discontiguous chain segments from the beta-sheet of Escherichia coli thioredoxin suggests an initiation
site for folding. Biochemistry.

[14] Bhutani N., Udgaonkar J. B. (2003). Folding
subdomains of thioredoxin characterized by
native-state hydrogen exchange. Protein Sci..

[15] Aslund F., Nordstrand K., Berndt K. D., Nikkola M., Bergman T., Ponstingl H., Jornvall H., Otting G., Holmgren A. (1996). Glutaredoxin
3 from Escherichia coli. Amino acid
sequence, 1H and 15N NMR assignments, and structural analysis. J. Biol. Chem..

[16] Pan J. L., Bardwell J. C. (2006). The origami of thioredoxin-like
folds. Protein Sci..

[17] Meyer Y., Buchanan B. B., Vignols F., Reichheld J. P. (2009). Thioredoxins
and glutaredoxins: Unifying elements in redox biology. Annu. Rev. Genet..

[18] Mondal S., Singh S. P. (2022). New insights on
thioredoxins (Trxs) and glutaredoxins
(Grxs) by *in silico* amino acid sequence, phylogenetic
and comparative structural analyses in organisms of three domains
of life. Heliyon.

[19] Nordstrand K., Slund F., Holmgren A., Otting G., Berndt K. D. (1999). NMR structure
of Escherichia coli glutaredoxin 3-glutathione
mixed disulfide complex: Implications for the enzymatic mechanism. J. Mol. Biol..

[20] Tran T. V., Hoang T., Jang S. H., Lee C. (2023). Unraveling the roles
of aromatic cluster side-chain interactions on the structural stability
and functional significance of psychrophilic *Sphingomonas* sp. glutaredoxin 3. PLoS One.

[21] Guo H.-B., Perminov A., Bekele S., Kedziora G., Farajollahi S., Varaljay V., Hinkle K., Molinero V., Meister K., Hung C. (2022). AlphaFold2
models indicate that protein sequence determines
both structure and dynamics. Sci. Rep..

[22] Shao C., Bittrich S., Wang S., Burley S. K. (2022). Assessing PDB macromolecular
crystal structure confidence at the individual amino acid residue
level. Structure.

[23] Meng E. C., Goddard T. D., Pettersen E. F., Couch G. S., Pearson Z. J., Morris J. H., Ferrin T. E. (2023). UCSF ChimeraX:
Tools for structure
building and analysis. Protein Sci.

[24] Madeira F., Madhusoodanan N., Lee J., Eusebi A., Niewielska A., Tivey A. R. N., Lopez R., Butcher S. (2024). The EMBL-EBI Job Dispatcher
sequence analysis tools framework in 2024. Nucleic
Acids Res..

[25] Gallivan J. P., Dougherty D. A. (1999). Cation-pi interactions in structural biology. Proc. Natl. Acad. Sci. U. S. A..

[26] Del
Conte A., Camagni G. F., Clementel D., Minervini G., Monzon A. M., Ferrari C., Piovesan D., Tosatto S. C. E. (2024). RING 4.0: Faster residue interaction networks with
novel interaction types across over 35,000 different chemical structures. Nucleic Acids Res..

[27] Huynh K., Partch C. L. (2015). Analysis of protein
stability and ligand interactions
by thermal shift assay. Curr. Protoc. Protein
Sci..

[28] Nick Pace, C. ; Martin Scholtz, J. , Measuring the conformational stability of a protein. In Protein Structure: A Practical Approach, 2nd ed.; IRL Press at Oxford University Press, 1997; pp. 299–321.

[29] Makhatadze G. I. (1999). Thermodynamics
of protein interactions with urea and guanidinium hydrochloride. J. Phys. Chem. B.

[30] Laws W. R., Contino P. B. (1992). Fluorescence quenching
studies: Analysis of nonlinear
Stern-Volmer data. Methods Enzymol..

[31] Micsonai A., Moussong É., Wien F., Boros E., Vadászi H., Murvai N., Lee Y. H., Molnár T., Réfrégiers M., Goto Y., Tantos Á., Kardos J. (2022). BeStSel: Webserver for secondary
structure and fold
prediction for protein CD spectroscopy. Nucleic
Acids Res..

[32] Heinig M., Frishman D. (2004). STRIDE: A web server for secondary structure assignment
from known atomic coordinates of proteins. Nucleic
Acids Res..

[33] Johansson C., Lillig C. H., Holmgren A. (2004). Human mitochondrial
glutaredoxin
reduces S-glutathionylated proteins with high affinity accepting electrons
from either glutathione or thioredoxin reductase. J. Biol. Chem..

[34] Begas P., Staudacher V., Deponte M. (2015). Systematic re-evaluation of the bis­(2-hydroxyethyl)­disulfide
(HEDS) assay reveals an alternative mechanism and activity of glutaredoxins. Chem. Sci..

[35] Lu C., Wu C., Ghoreishi D., Chen W., Wang L., Damm W., Ross G. A., Dahlgren M. K., Russell E., Von Bargen C. D., Abel R., Friesner R. A., Harder E. D. (2021). OPLS4: Improving
force field accuracy on challenging regimes of chemical space. J. Chem. Theory Comput..

[36] Frank H. S., Wen W.-Y. (1957). Ion-solvent interaction.
Structural aspects of ion-solvent
interaction in aqueous solutions: A suggested picture of water structure. Discuss. Faraday Soc..

[37] Vazquez D. S., Delfino J. M., Santos J. (2015). Thioredoxin from Escherichia
coli as a role model of molecular recognition, folding,
dynamics and function. Protein Pept. Lett..

[38] Qi Y., Grishin N. V. (2005). Structural classification of thioredoxin-like fold
proteins. Proteins.

[39] Ren G., Stephan D., Xu Z., Zheng Y., Tang D., Harrison R. S., Kurz M., Jarrott R., Shouldice S. R., Hiniker A., Martin J. L., Heras B., Bardwell J. C. (2009). Properties
of the thioredoxin fold superfamily are modulated by a single amino
acid residue. J. Biol. Chem..

[40] Katti S. K., LeMaster D. M., Eklund H. (1990). Crystal structure
of thioredoxin
from Escherichia coli at 1.68 A resolution. J. Mol. Biol..

[41] Napolitano S., Reber R. J., Rubini M., Glockshuber R. (2019). Functional
analyses of ancestral thioredoxins provide insights into their evolutionary
history. J. Biol. Chem..

[42] Tasayco M. L., Chao K. (1995). NMR study of the reconstitution
of the beta-sheet of thioredoxin
by fragment complementation. Proteins.

[43] Nguyen T.-T., Hoang T., Tran K. N., Kim H., Jang S.-H., Lee C. (2021). Essential roles of buried phenylalanine in the structural stability
of thioredoxin from a psychrophilic Arctic bacterium *Sphingomonas* sp. PLoS One.

[44] Assemat K., Alzari P. M., Clement-Metral J. (1995). Conservative
substitutions in the
hydrophobic core of *Rhodobacter sphaeroides* thioredoxin
produce distinct functional effects. Protein
Sci..

[45] Nordstrand K., Sandström A., Aslund F., Holmgren A., Otting G., Berndt K. D. (2000). NMR structure of oxidized glutaredoxin 3 from Escherichia coli. J. Mol. Biol..

[46] Van
Tran T., Nguyen H., Vu L., Lee C. (2024). Structural conservation
in the glutathione binding in *Sphingomonas* sp. glutaredoxin
Grx3 and variations for cold adaptation. Biochim.
Biophys. Acta, Proteins Proteomics.

[47] Dougherty
Dennis D. A. (2007). Cation-π interactions involving aromatic amino
acids. J. Nutr..

[48] Scherer S. L., Stewart A. L., Fortenberry R. C. (2018). Patterns
of cation binding to the
aromatic amino acid R groups in Trp, Tyr, and Phe. Comput. Biol. Chem..

[49] Wang Y., Hou Y., Wang Q. (2021). Cloning, expression,
characterization, and antioxidant
protection of glutaredoxin3 from psychrophilic bacterium *Psychrobacter* sp. Ant206. Front. Microbiol..

[50] van
Dijk E., Hoogeveen A., Abeln S. (2015). The hydrophobic temperature dependence
of amino acids directly calculated from protein structures. PLoS Comput. Biol..

[51] Kashif A., Tran L. H., Jang S. H., Lee C. (2017). Roles of active-site
aromatic residues in cold adaptation of *Sphingomonas glacialis* esterase EstSP1. ACS Omega.

[52] Hoang T., Jeong C., Jang S. H., Lee C. (2023). Tyr76 is essential
for the cold adaptation of a class II glutaredoxin 4 with a heat-labile
structure from the Arctic bacterium *Sphingomonas* sp. FEBS Open Bio.

