# Supplementary material for: Aromatic Residue Variations in the Central β‑Sheet Influence Stability and Activity of E. coli Glutaredoxin 3
Source: ACS Omega. 2025 Jun 9;10(24):25810–8. doi: 10.1021/acsomega.5c01938 (PMC12199019; doi:10.1021/acsomega.5c01938)
Supplement: Supplementary file 1 [file ao5c01938_si_001.pdf]

Supporting information for:

Aromatic Residue Variations in the Central β-Sheet Influence Stability and Activity of *E. coli* Glutaredoxin 3

Mohammed Shazaly A. Elhassan,[1] Trang Van Tran,[1,2] ChangWoo Lee [1,*]

[1]Department of Biomedical Science and Center for Bio-Nanomaterials, Daegu University, Gyeongsan 38453, South Korea
[2]Phacogen Institute of Technology, Hanoi 10700, Vietnam

Table S1. List of primers for site-directed mutagenesis.

	Primer sequences	
Y6F	Forward	5′-gcaacgttgaaataTTCactaa-3′
	Reverse	5′-ggtttctttagtGAAtatttcaac-3′
F56A	Forward	5′-cggtaccgcagattGCTattg-3′
	Reverse	5′-caatAGCaatctgcggtaccg-3′
F56E	Forward	5′-cgcagattGAGattgatgcacag-3′
	Reverse	5′-ctgtgcatcaatCTCaatctgcg-3′
F56I	Forward	5′-gtaccgcagattATTattgat-3′
	Reverse	5′-ctgtgcatcaatAATaatctg-3′
F56S	Forward	5′-ccgcagattAGTattgatgcacagc-3′
	Reverse	5′-gctgtgcatcaatACTaatctgcgg-3′
F56W	Forward	5′-cgcagattTGGattgatg-3′
	Reverse	5′-catcaatCCAaatctgcg-3′
F56Y	Forward	5′-gtaccgcagattTATattgat-3′
	Reverse	5′-ctgtgcatcaatATAaatctg-3′

Mutated nucleotides are shown in capital letters. The Y6F/F56Y double mutant was constructed using the Y6F primers with the F56Y mutant as the template.

Table S2. Stability parameters for EcGrx3 WT and mutants.

	$[D]_{½}$ [a]	*m* [b]	$\Delta G^{0\prime}_{H_2O}$ [c]
	(M)	(kcal mol^{-1} M^{-1})	(kcal mol^{-1})
WT	3.3 ± 0.06	1.08 ± 0.01	3.58 ± 0.07
F56A	2.6 ± 0.06	0.89 ± 0.02	2.61 ± 0.03
F56E	1.7 ± 0.02	0.72 ± 0.02	1.32 ± 0.03
F56I	2.3 ± 0.07	0.79 ± 0.04	1.85 ± 0.01
F56S	2.1 ± 0.03	0.75 ± 0.04	1.73 ± 0.05
F56Y	2.9 ± 0.08	0.82 ± 0.05	2.41 ± 0.10
F56W	1.3 ± 0.01	0.64 ± 0.01	0.89 ± 0.01
Y6F/F56Y	1.9 ± 0.04	0.70 ± 0.03	1.57 ± 0.07

[a] GdmCl concentration at the midpoint of the unfolding transition.

[b] Proportionality constant relating free energy to GdmCl concentration.

[c] Free energy of unfolding extrapolated to 0 M denaturant.

Data are presented as the mean ± S.D. from three independent measurements.

Table S3. Inverse Stern–Volmer quenching constant K_{sv}^{-1} for EcGrx3 WT and mutants.

	K_{SV}^{-1} (mM)
WT	46 ± 2
F56A	28 ± 1
F56E	31 ± 3
F56I	30 ± 4
F56S	23 ± 3
F56W	67 ± 8
F56Y	44 ± 3
Y6F/F56Y	33 ± 4

K_{sv}^{-1} represents the acrylamide concentration at which 50% of the fluorescence intensity is quenched.

Table S4. Secondary structure composition of EcGrx3 WT and mutants.

	α-Helix (%)	β-Strand (%)	Turn (%)	Other (%)
WT	50	22	28	0
Y6F	8	20	17	55
F56A	35	29	2	34
F56E	10	17	13	60
F56I	34	32	4	30
F56S	18	21	7	54
F56W	57	43	0	0
F56Y	42	38	0	20
Y6F/F56Y	18	21	6	55

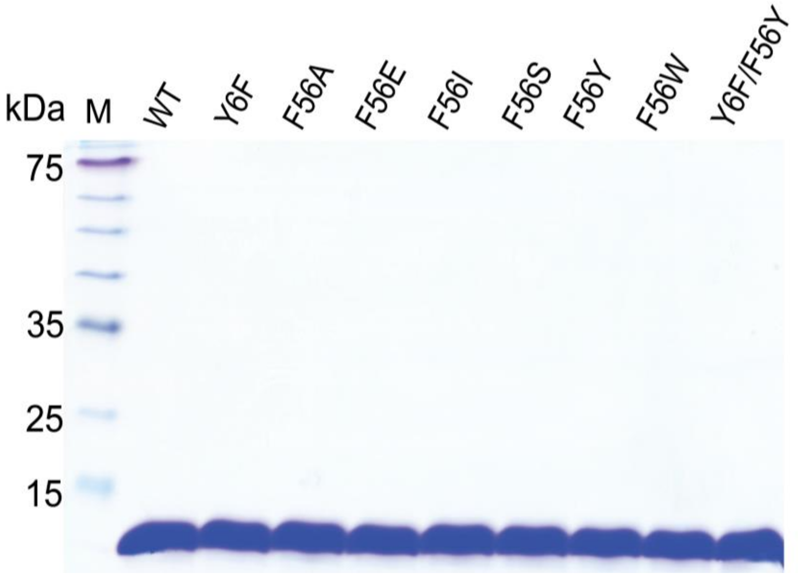

Figure S1. SDS-PAGE analysis of EcGrx3 WT and mutants. M, marker.

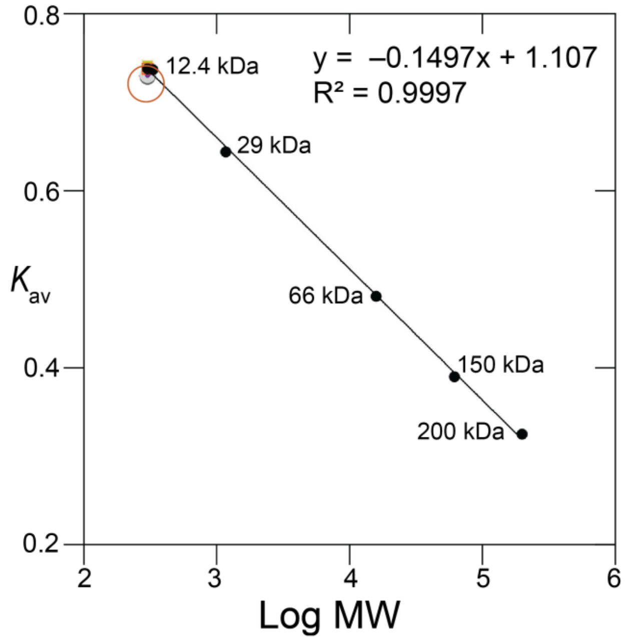

Figure S2. Size-exclusion chromatography analysis. The molecular weights (MWs) of EcGrx3 WT and its mutants were determined by size-exclusion chromatography using a Superdex 200 column. The circle indicates the estimated MW of EcGrx3 WT (12.5 kDa). The protein standard mix included β-amylase (200 kDa), alcohol dehydrogenase (150 kDa), albumin (66 kDa), carbonic anhydrase (29 kDa), and cytochrome c (12.4 kDa).

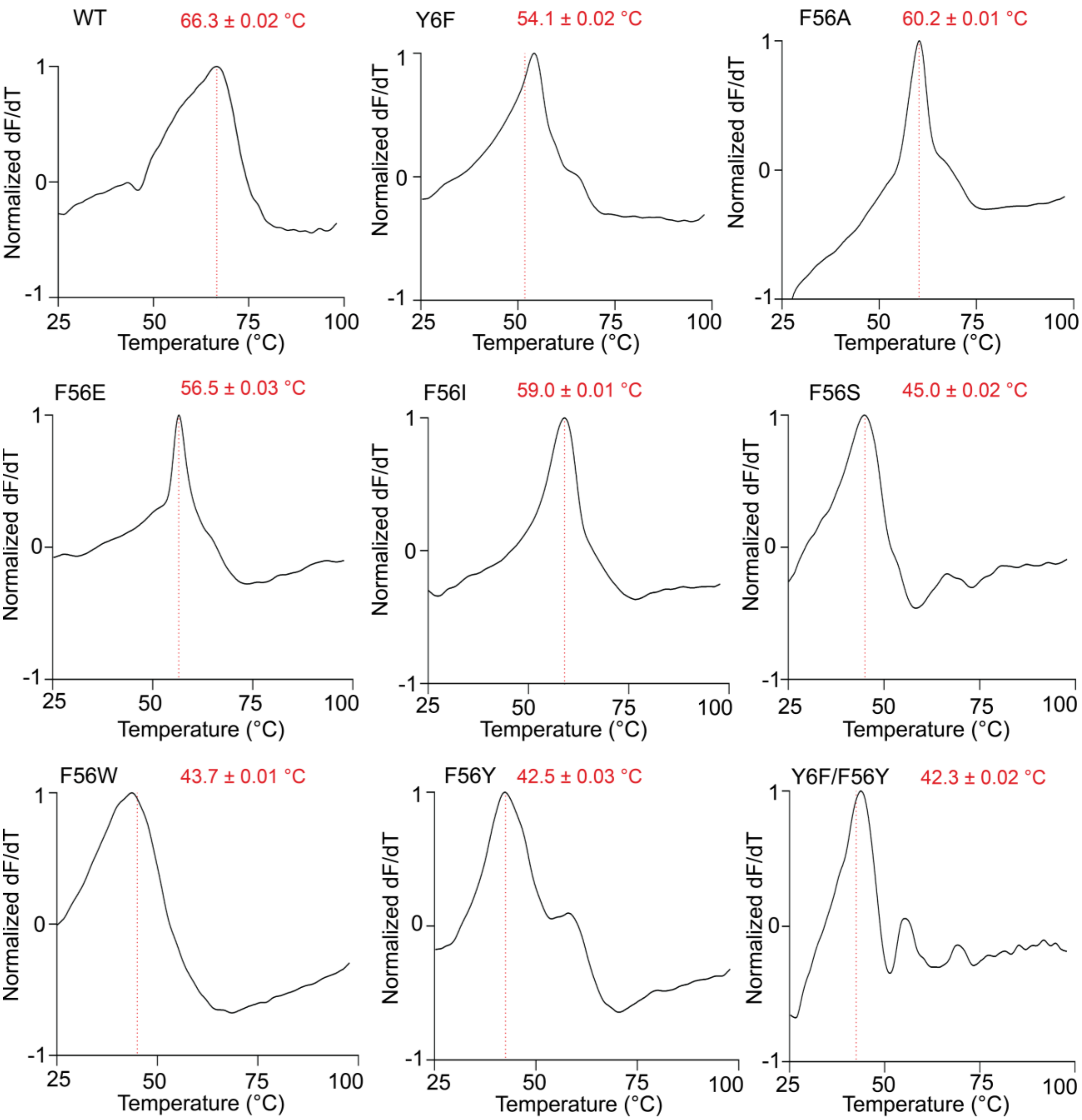

Figure S3. Melting temperatures of EcGrx3 WT and mutants. Melting temperatures were measured using SYPRO Orange-based thermal shift analysis from 25 to 99 °C with a continuous temperature ramp of 1 °C per min. The derivative dF/dT represents the rate of change in fluorescence signal (F) with respect to temperature (T) during thermal denaturation. Red dotted vertical lines indicate the peak of the derivative curve, corresponding to the thermal unfolding midpoint (T_m). Fluorescence was normalized by setting the maximal signal to 1. Data are presented as the mean ± S.D. of three experiments.

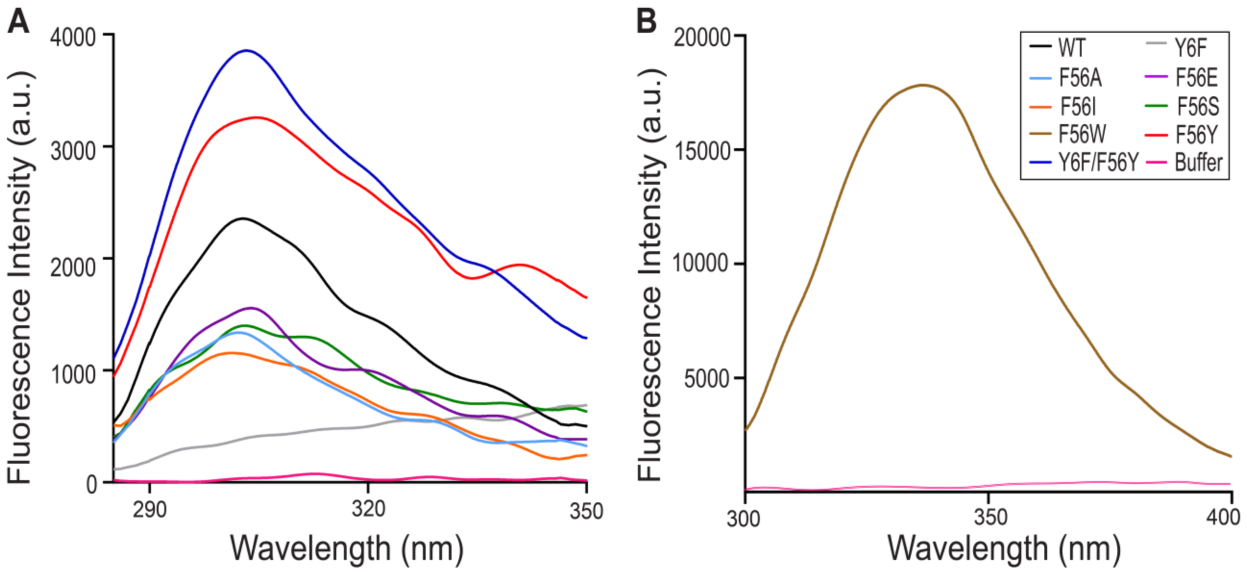

Figure S4. Intrinsic fluorescence of EcGrx3 WT and mutants at 25 °C. (A) Spectra of WT and mutants except F56W (excitation at 275 nm). (B) Spectra of F56W (excitation at 285 nm).

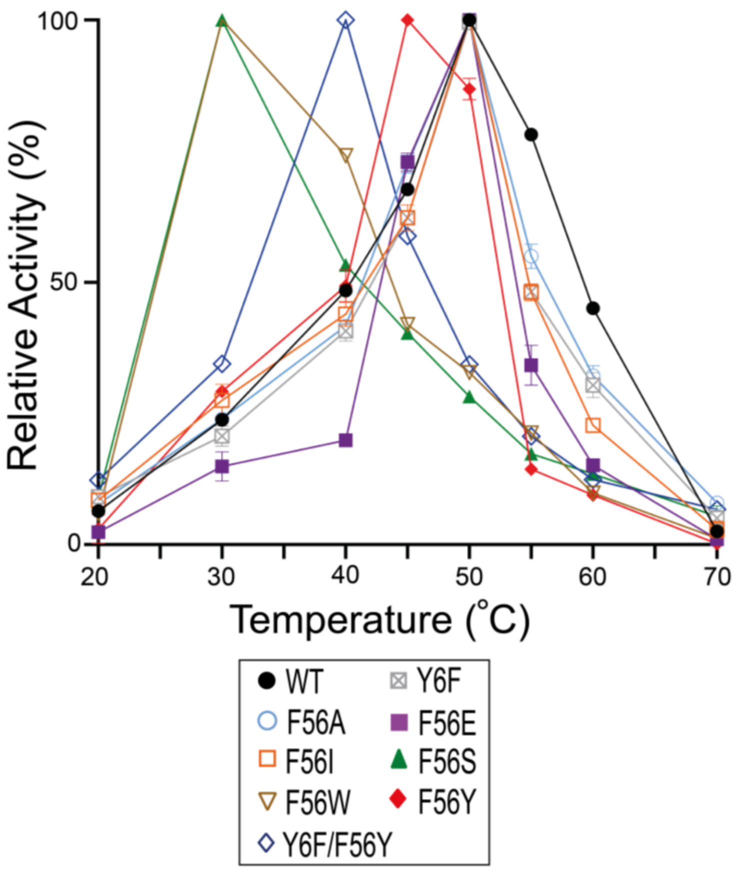

Figure S5. Apparent optimal temperatures of EcGrx3 WT and mutants. The activity at each protein's optimal temperature was set to 100%. Data are presented as the mean ± S.D. of three experiments.

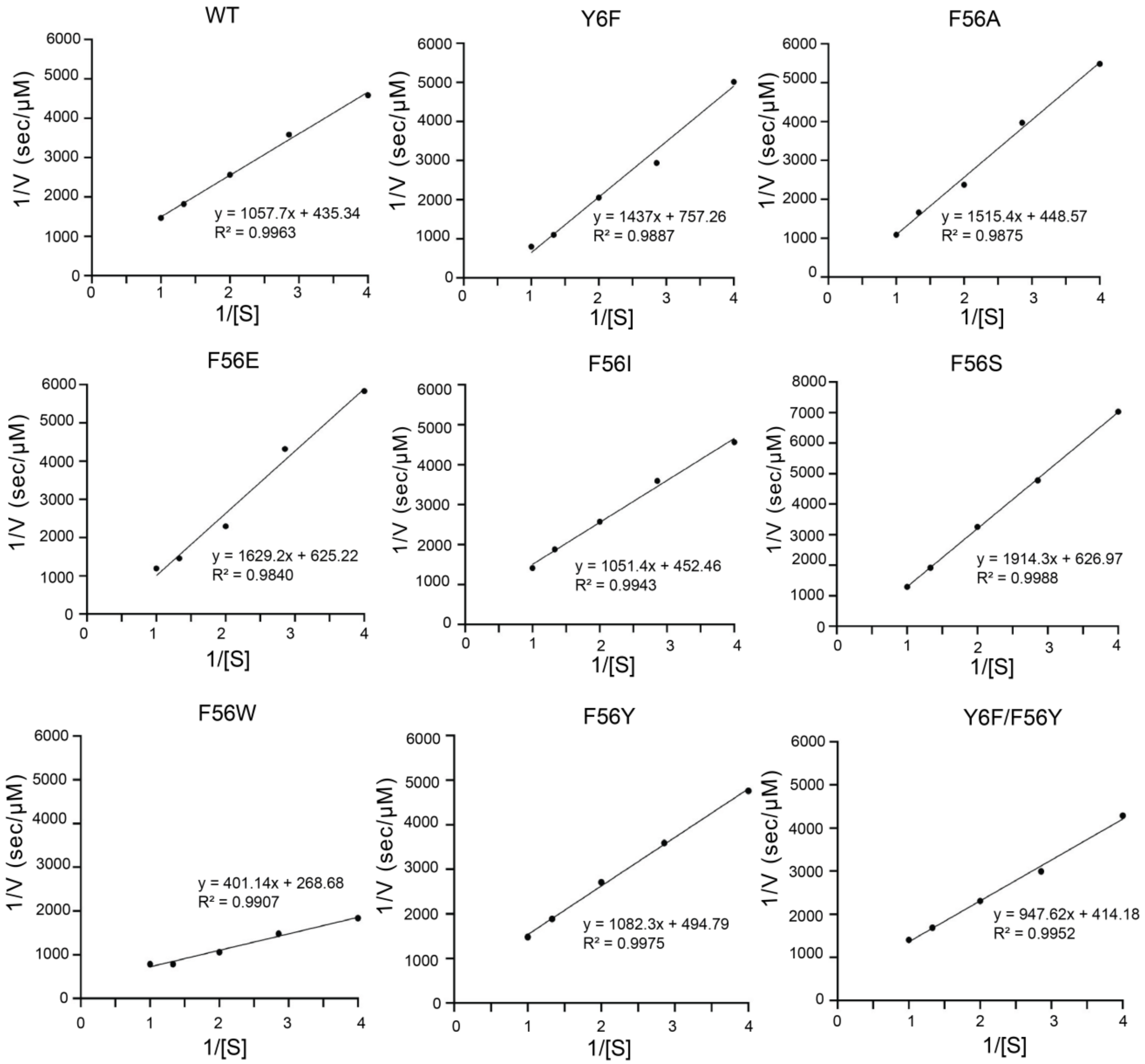

Figure S6. Lineweaver–Burk plots of EcGrx3 WT and mutants. Initial reaction velocities were determined by monitoring the decrease in NADPH absorbance at 340 nm over 5 min with GSH concentrations of 0.25–1.0 mM. Lineweaver–Burk plots (1/V vs. 1/[S]) were used to calculate apparent K_m and V_{max} values by linear regression, and k_{cat} was obtained by dividing V_{max} by the total enzyme concentration ([Et]).

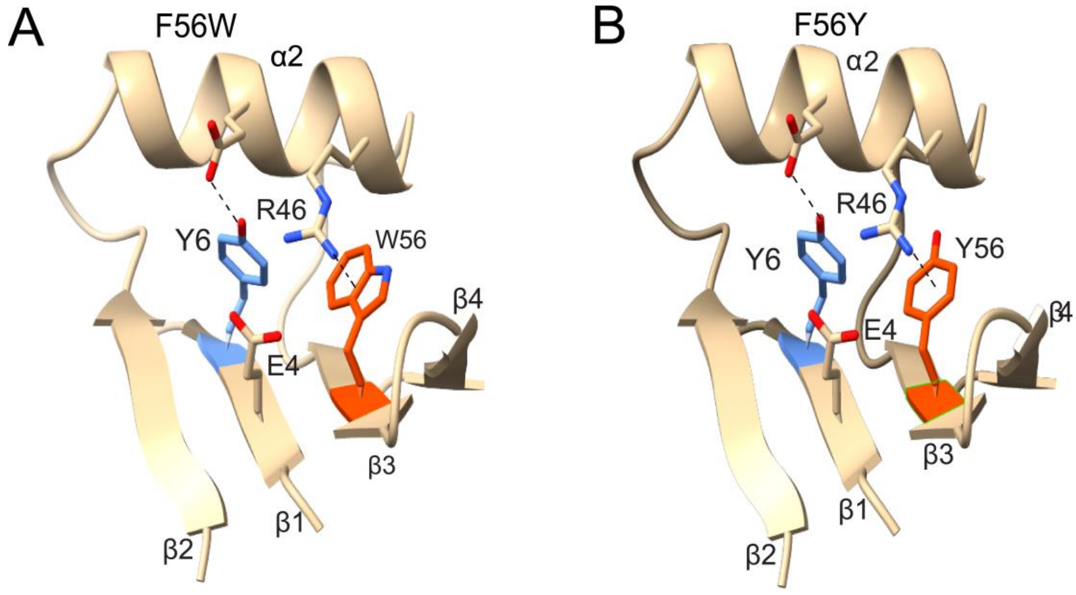

Figure S7. Cation-π interactions of F56W and F56Y mutants. AlphaFold2-predicted structures of F56W (A) and F56Y (B) were visualized and analyzed using ChimeraX (version 1.9).

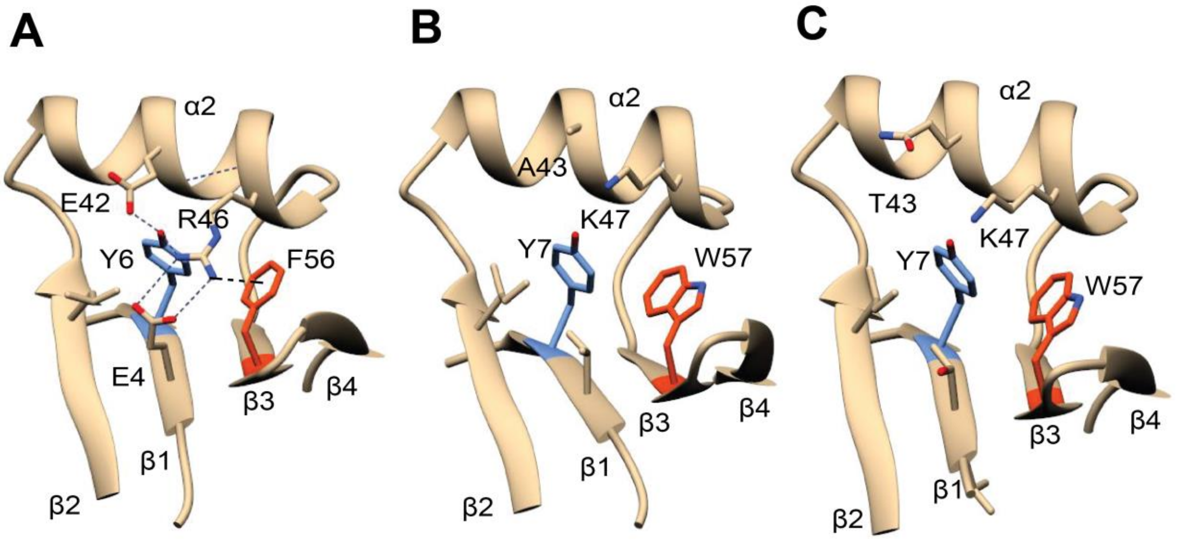

Figure S8. Structural comparison of Grx3 orthologs. (A) Mesophilic EcGrx3, (B) psychrotrophic PmGrx3, and (C) psychrophilic MpGrx3. The crystal structure of EcGrx3 (PDB ID: 1FOV) and AlphaFold2-predicted structures of PmGrx3 and MpGrx3 were visualized and analyzed using ChimeraX software (version 1.9).
